# Imputation in families using a heuristic phasing approach

**DOI:** 10.1186/1753-6561-8-S1-S16

**Published:** 2014-06-17

**Authors:** August N Blackburn, Angela K Dean, Donna M Lehman

**Affiliations:** 1 Department of Cellular and Structural Biology, University of Texas Health Science Center at San Antonio, 7702 Floyd Curl Road, San Antonio, TX 78229, USA; 2 Department of Medicine/Clinical Epidemiology, University of Texas Health Science Center at San Antonio, 7702 Floyd Curl Road, San Antonio, TX 78229, USA

## Abstract

Whole genome sequencing (WGS) remains prohibitively expensive, which has encouraged the development of methods to impute WGS data into nonsequenced individuals using a framework of single nucleotide polymorphisms genotyped for genome-wide association studies (GWAS). Although successful methods have been developed for cohorts of unrelated individuals, current imputation methods in related individuals are limited by pedigree size, by the distance of relationships, or by computation time. In this article, we describe a method for imputation in arbitrarily shaped multigenerational pedigrees that can impute genotypes across distantly related individuals based on identity by descent. We evaluate this approach using GWAS data and apply this approach to WGS data distributed for Genetic Analysis Workshop 18.

## Background

Recent years have seen a sharp increase in the throughput of genotyping in human cohorts represented largely by two technologies: microarray-based approaches that genotype hundreds of thousands of markers tagging common haplotype blocks for the purpose of genome-wide association studies (GWAS) and WGS, which allows near comprehensive discovery of genotypes. Many groups have the resources to genotype entire cohorts for GWAS; however, WGS currently remains prohibitively expensive. In light of this, imputation of WGS data from reference panels using available GWAS data has been adopted as an approach to maximize the utility of available resources [1].

Merlin [2] and Mendel [3] are two programs that have built-in options for genotype imputation designed for working with pedigrees. However, these two methods have limited ability to handle large pedigrees and do not impute variants across more distantly related individuals in the same pedigree. Markov chain Monte Carlo (MCMC) methods that sample across potential identity-by-descent (IBD) states are an approach that makes broader use of the information contained between more distant relatives but are still currently being developed as an imputation approach [4]. However, this approach requires iterative sampling that is computationally expensive and may limit its utility in laboratories with limited computing resources. An ideal imputation approach would be highly accurate and computationally fast and would consider all chromosomal segments that are identical by descent within the pedigree.

In this article, we describe and evaluate a computationally feasible rules-based approach for phasing and imputation that can impute across distantly related individuals within complex pedigrees.

## Methods

### Data handling and marker location annotation

WGS data was managed using VCFtools [5]. GWAS marker locations for NCBI Build GRCh37/hg19 were annotated using SNPnexus [6].

### Assumptions of our imputation approach

Our approach requires two sets of genotyping data. The first set (set 1), generally GWAS SNP data, is used to identify chromosomal segments that are IBD. The second set (set 2), either dense GWAS or WGS data, consists of variants that are incompletely genotyped in individuals with set 1 data. Using identity by descent, set 2 genotypes are imputed into individuals with missing genotypes. Our approach requires correct knowledge of the pedigree structure and all individuals to have set 1 data. Our approach breaks pedigrees containing individuals without set 1 data into smaller pedigrees with complete data.

### **Phasing pedigrees using the genome-wide association studies framework**

**Figure 1:**
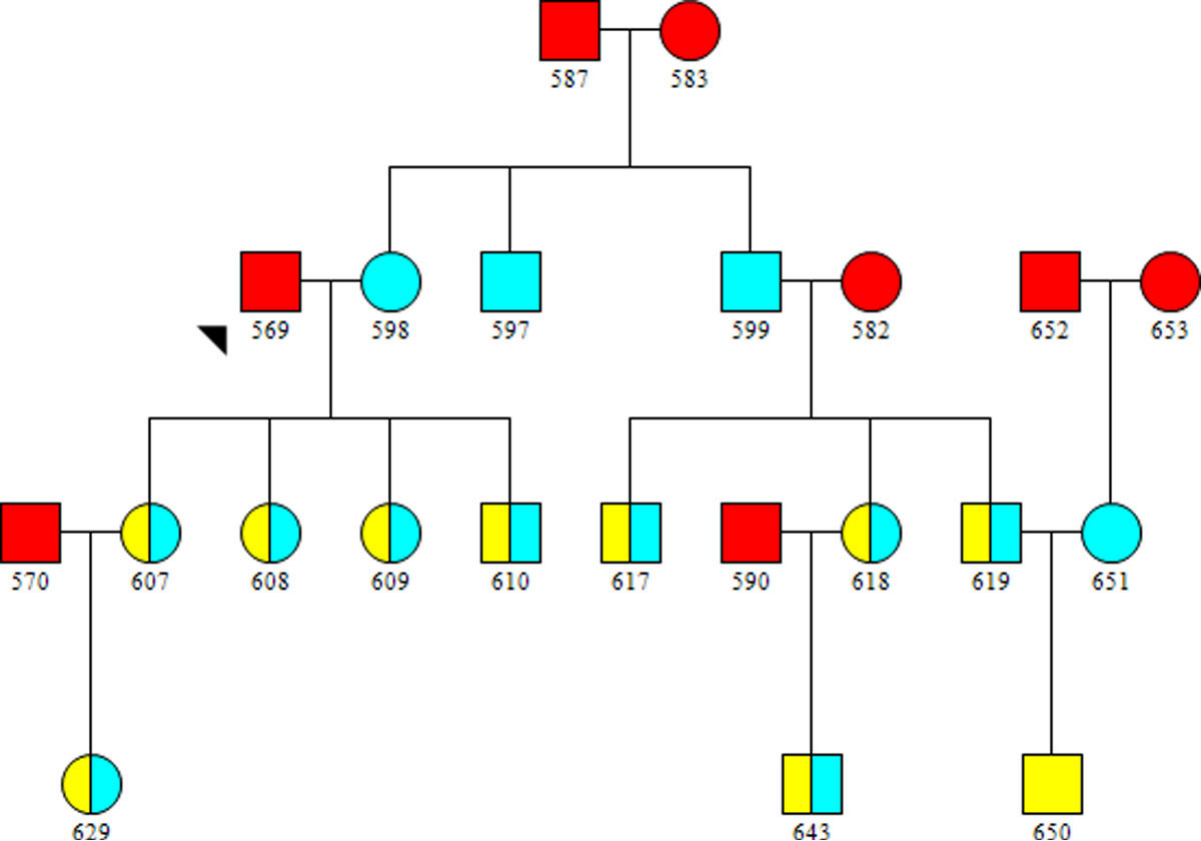
**An example pedigree partially simulated from pedigree 10 from Genetic Analysis Workshop 18 data**. Founders are shown in red, individuals carrying nonfounding chromosomes that are the product of a single meiosis are shown in blue, and individuals carrying nonfounding chromosomes that are products of two or more meioses are shown in yellow.

Using the GWAS framework (set 1), we first find all available trios in the pedigree and phase the offspring and parents according to rules of inheritance for diallelic markers. In our view, two subtypes of nonfounder chromosomes lend themselves to distinct approaches for phasing and identification of recombination events: chromosomes that are the product of a single round of meiosis within the pedigree and chromosomes that are the product of two or more rounds of meiosis within the pedigree. It is important to note that nonfounders can carry both of the aforementioned subtypes of nonfounder chromosomes as illustrated in Figure 1.

Cross-over events for nonfounder chromosomes that have experienced two or more meioses within the pedigree can be determined using rules of inheritance for diallelic markers. To implement this approach, we find all cases in which a parent in a trio is an offspring in another trio such as ID 651 in Figure 1, who is the offspring in a trio with 652 and 653, and a parent in a trio with 619 and 650. The exact phase of 651 and 650 is known from the trio phasing stage. With this information, each phased locus where 651 is heterozygous represents an unambiguous instance where the genotype that 650 inherited from 651 is identical by descent to a genotype from either 652 or 653. We applied this concept to identify the recombination events between informative markers and stored the IBD segments between the recombination events and their 3-generation paths of inheritance.

All other nonfounder chromosomes in the pedigree are the result of recombination events during a single meiosis within the pedigree and are only carried by offspring of a pedigree founder. For this subset of nonfounder chromosomes, cross-over events cannot be determined using rules of inheritance as they can for those just discussed. Within this subset of nonfounder chromosomes, we applied a strategy aimed at accomplishing two goals: (a) to identify observable recombination events in the offspring and (b) to create a representation of the founder's phase that is as close to their true phase as possible and is consistent with the observable recombination events in their offspring. To accomplish this task, we implemented a version of the minimum recombination model [7]. The exact phase of founder offspring is known from trio phasing. Given this information, markers that are heterozygous in the founder and phased in their offspring can be dichotomized into identical by descent groups corresponding to the two haploid genotypes carried by the founder. Multiple markers along long segments of the chromosome show consistent dichotomized groups. Instances where the members of these groups change between adjacent informative markers indicate the location of a recombination event. Our model assumes that the minimum number of recombination events needed to explain the change in dichotomized groups represents the true number of recombination events. In cases in which a founder has two offspring, it is possible to identify recombination events, but it cannot be determined which offspring harbors the recombination event, as elegantly shown by Roach *et al *[8]. For these cases, we arbitrarily assign the recombination event to one of the offspring. Last, for founders with a single offspring, we use the phased chromosome inherited by the offspring as a proxy for the true phase of the founder. This approach allows us to create a single representation of the founder's phase that fits the identifiable recombination events in their offspring without violating any rules of inheritance. After applying these approaches, we store the IBD segments between the observable recombination events and their corresponding 2-generation paths of inheritance.

### Identifying exact paths of inheritance for all identity-by-descent segments

Using the approach for phasing outlined, all individuals with set 1 genotypes will be phased. Two-generation or 3-generation paths of inheritance for all IBD segments of each phased chromosome will be known. We use the available 2-generation and 3-generation paths of inheritance to trace the IBD segments to an exact phased founder chromosome. To do this, we implement a recursive algorithm that traverses all potential paths of inheritance from each founding chromosome. During each step representing a meiosis traversed by the algorithm, segments inherited from the current founding chromosome are passed on to the next generation according to their overlap with segments discovered to have been inherited along the current path. The end result is a list of IBD segments and their paths of inheritance, which summarizes the genotypes observed in nonfounders in terms of the founding chromosomes. We store this information as a list of IBD segments and their paths of inheritance, which we call a nonfounder matrix.

### Diploid genotype imputation

We then leverage the IBD information and paths of inheritance stored in the nonfounder matrix to impute genotypes among individuals within a pedigree. To do this, we first phase all set 2 genotypes possible using trio phasing and by splitting homozygous genotypes. In doing so, we unambiguously map each haploid genotype to a specific IBD segment stored in the nonfounder matrix. We then use the paths of inheritance for each IBD segment stored in the nonfounder matrix to impute these haploid genotypes into the founding chromosomes. If a sequenced founder is heterozygous for a marker and only one haploid genotype could be imputed onto a specific phased chromosome, we infer the corresponding haploid genotype on the paired chromosome given the sequenced diploid genotype. In any individual within the pedigree, a diploid genotype can be accounted for by the two haploid genotypes inherited from pedigree founders. Diploid genotypes are then imputed by summing the two haploid genotypes identical by descent to founding chromosomes as recorded in the nonfounder matrix. As an example, using these procedures, a haploid genotype on an IBD segment shared between 650 and 583 that is genotyped and phased in 650 could be imputed into 629 if 629 also shares a segment IBD to 583 at that locus.

## Results

To estimate the accuracy of the described method, we split the provided GWAS data evenly into two subsets for use as set 1 and set 2 genotyping data. Fifty percent of set 2 genotypes were randomly masked from the imputation procedures. The outlined procedures were followed, and IQS [9], a statistic that corrects for random concordance between imputed and known genotypes, was calculated by comparing the imputed genotypes to the masked genotypes. The mean IQS was 0.992 for 211,736 markers (18,498,441 imputed diploid genotypes) for which the chance concordance was less than 1. This high accuracy was robust across the minor allele frequency spectrum, calculated using the set of 157 unrelated individuals provided by the data distributors. For markers with MAF in the ranges 0 to 0.01, greater than 0.01 to 0.05, greater than 0.05 to 0.1, greater than 0.1 to 0.2, greater than 0.2 to 0.3, greater than 0.3 to 0.4, and greater than 0.4 to 0.5 the mean IQS was 0.972, 0.985, 0.990, 0.992, 0.993, 0.994, and 0.994, respectively. To assess the robustness of this approach to the availability of set 2 data, we repeated this masking experiment, this time randomly masking genotypes using a randomly generated probability ranging from 0 to 1 for each marker. This test showed that the accuracy of the described imputation approach is robust across levels of available data. The overall mean IQS for this experiment was 0.991 for 187,793 markers (11,242,219 imputed diploid genotypes) for which the chance concordance was less than 1. For markers with 0 to 0.1, greater than 0.1 to 0.25, greater than 0.25 to less than 0.75, 0.75 to less than 0.9, and less than 0.9 to 1 of diploid genotypes available, the mean IQS across markers was 0.984, 0.988, 0.992, 0.993, and 0.994, respectively. The percentage of the masked genotypes that this approach was able to impute was positively correlated with the percentage of set 2 genotypes that were known.

To assess the utility of this approach in a real-world application, we used the provided GWAS data as set 1 and the provided WGS data as set 2. Using the procedures described herein, we imputed WGS data for all odd-numbered autosomes into 408 individuals (227 with sequence data and 181 without sequence data) from 16 pedigrees containing sequenced individuals. In individuals who were not sequenced, we imputed 1.66 × 10^9 ^and 1.09 × 10^8 ^homozygous and heterozygous genotypes, respectively, an average of 9.17 × 10^6 ^and 6.00 × 10^5 ^imputed homozygous and heterozygous genotypes per individual, respectively. This represents a 132% return on investment per sequenced diploid genotype in these pedigrees.

## Discussion

We have developed a method for imputing WGS data in arbitrarily shaped pedigrees using a GWAS framework. As a step toward achieving this, we have developed a heuristic method to phase whole chromosomes in arbitrarily shaped multigenerational pedigrees. Our approach differs from previous approaches in that it organizes chromosomes according to the number of rounds of meiosis they have experienced in the pedigree and implements distinct strategies for phasing and identification of recombination events for these chromosomes.

In this study, we applied no filters for markers that display genotyping errors in trios, have de novo mutations within the pedigree or overlap copy number variable regions. When comparing imputed WGS data with known genotypes, 61,360 of 700,423 (8.8%) markers with at least one incorrect imputed diploid genotype fell within copy number variable regions previously identified in individuals within the GAW18 dataset [10], a clear enrichment compared to the 6.8% expected to fall into these regions by chance based on the portion of these chromosomes that were variable on a per nucleotide basis (*p *= 2.47 × 10^−323^, two-sided binomial test). More strikingly, 37.4% fell within regions reported in the Database of Genomic Variants [11]. Therefore, evidence suggests that this approach would be improved by incorporating structural variation.

Our method requires all individuals within a pedigree to have set 1 genotyping data. Given this, MCMC methods are likely to be superior for imputation in pedigrees with many or key missing individuals. However, in pedigrees with complete set 1 data, the method described herein imputes across all segments that are identical by descent with nearly perfect accuracy. The described masking experiment ran in 7 minutes for chromosome 1 in pedigree 47 on a Lenovo ThinkStation S20 with an Intel Xeon 2.40 GHz CPU. All procedures described in this manuscript were run on this same machine. Thus, this approach is computationally feasible for small laboratories. Taken together, in pedigrees with complete data, the described method is highly accurate and computationally feasible and considers all chromosomal segments that are identical by descent within the pedigree.

## Conclusions

We have developed an accurate and computationally feasible method for phasing and imputation in large arbitrarily shaped multigenerational pedigrees. In pedigrees with a complete GWAS framework data, this method is able to impute genotypes across all IBD segments within the pedigree. Our results show that an imputation strategy based on genetic phasing of GWAS data can be a successful approach to imputation in multigenerational pedigrees.

## Competing interests

The authors declare that they have no competing interests.

## Authors' contributions

ANB conceived of the phasing, storage, and imputation procedures and designed the overall study. ANB and AKD implemented the ideas using the perl scripting language. ANB, AKD, and DML all contributed to the development of the procedures described. ANB and DML drafted the manuscript. All authors read and approved the final manuscript.
